# A novel mutatioin in the *PSTPIP1* gene is associated with an autoinflammatory disease distinct from classical PAPA syndrome

**DOI:** 10.1186/1546-0096-9-S1-O39

**Published:** 2011-09-14

**Authors:** D Holzinger, J Austermann, P Lohse, I Aksentijevich, S Holland, M Gattorno, C Rodríguez-Gallego, S Fessatou, B Isidor, S Tokio, J Bernstein, B Sampson, C Sunderkoetter, J Roth

**Affiliations:** 1 Institute of Immunology, University Muenster, Muenster, Germany; 2 Department of Clinical Chemistry Großhadern, University Munich, Munich, Germany; 3 National Institute of Arthritis and Musculoskeletal and Skin Diseases, Bethesda, ,USA; 4 National Institute of Allergy and Infectious Diseases, Bethesda, USA; 5 2nd Division of Pediatrics "G. Gaslini" Scientific Institute, Genova, Italy; 6 Department of Immunology, Gran Canaria Dr. Negrín University Hospital, Las Palmas de Gran Canaria, Spain; 7 Department of Pediatrics , Attikon Hospital, Athens, Greece; 8 Service de Génétique Médicale, Centre Hospitalo-Universitaire, Nantes, France; 9 Nagoya City University, Tokyo, Japan; 10 Department of Pediatrics, Stanford University Medical Center, Stanford, USA; 11 Department of Clinical Chemistry, Charing Cross Hospital, London, UK; 12 Department of Dermatology, University Hospital Muenster, Muenster, Germany

## Background

Hyperzincaemia and hypercalprotectinaemia, a rare condition within the spectrum of autoinflammatory diseases, is associated with recurrent infections, hepatosplenomegaly, arthritis, anemia, cutaneous inflammation, and failure to thrive. So far, no genetic cause has been identified in these patients. While the clinical appearance is heterogeneous, all affected individuals present with extremely elevated S100A8/S100A9 (calprotectin) serum concentrations (0.9-12.0 g/l (normal range < 0.001 g/l)).

## Aim

The clinical phenotype of 8 patients was characterized. Screening of candidate genes *PSTPIP1* and *MEFV* was performed in 7 hyperzincaemia and hypercalprotectinaemia patients to identify disease-causing mutations.

## Methods

Serum concentrations of S100A8/S100A9 were analyzed by an ELISA assay in 8 patients with hyperzincaemia and hypercalprotectinaemia and compared to PAPA patients with and without treatment. Candidate exons were amplified by PCR and sequenced on an ABI 3130 Genetic Analyzer.

## Results

Seven of the eight patients were heterozygous carriers of a glutamic acid 250 (GAG)→lysine (AAG)/p.Glu250Lys/E250K substitution in exon 11 of the *PSTPIP1* gene. S100A8/S100A9 concentrations were extremely elevated in these patients (0.9-12 g/l) compared to seven patients presenting with classical PAPA symptoms (0.02-0.35 g/l), whose levels again were significantly higher compared to normal controls.

## Conclusion

The PSTPIP1 E250K mutation causes an autoinflammatory disorder known as hyperzincaemia and hypercalprotectinaemia. The disease causes a heterogeneous spectrum of symptoms that only partially overlaps with the presentation of the classical PAPA syndrome. Elevated S100A8/A9 levels are a common hallmark and biomarker of disorders caused by mutations in the *PSTPIP1* gene.

